# Alterations in Cerebral Blood Flow after Resuscitation from Cardiac Arrest

**DOI:** 10.3389/fped.2017.00174

**Published:** 2017-08-16

**Authors:** Bistra Iordanova, Lingjue Li, Robert S. B. Clark, Mioara D. Manole

**Affiliations:** ^1^Department of Bioengineering, University of Pittsburgh, Pittsburgh, PA, United States; ^2^School of Pharmacy, University of Pittsburgh, Pittsburgh, PA, United States; ^3^Safar Center for Resuscitation Research, Department of Pediatrics, University of Pittsburgh, Pittsburgh, PA, United States; ^4^Safar Center for Resuscitation Research, Department of Critical Care Medicine, University of Pittsburgh, Pittsburgh, PA, United States

**Keywords:** cerebral perfusion, cerebral blood flow, cardiac arrest, post-cardiac arrest syndrome, transcrianial Doppler, arterial spin labeling, hypoperfusion, hyperemia

## Abstract

Greater than 50% of patients successfully resuscitated from cardiac arrest have evidence of neurological disability. Numerous studies in children and adults, as well as in animal models have demonstrated that cerebral blood flow (CBF) is impaired after cardiac arrest. Stages of cerebral perfusion post-resuscitation include early hyperemia, followed by hypoperfusion, and finally either resolution of normal blood flow or protracted hyperemia. At the level of the microcirculation the blood flow is heterogeneous, with areas of no flow, low flow, and increased flow. CBF directed therapies in animal models of cardiac arrest improved neurological outcome, and therefore, the alterations in CBF after cardiac arrest likely contribute to the development of hypoxic ischemic encephalopathy. Current intensive care after cardiac arrest is centered upon maintaining systemic oxygenation, normal blood pressure values for age, maintaining general homeostasis, and avoiding hyperthermia. Assessment of CBF and oxygenation is not routinely performed after cardiac arrest. Currently available and underutilized techniques to assess cerebral perfusion include transcranial doppler, near-infrared spectroscopy, and arterial spin labeling magnetic resonance imaging. Limited clinical studies established the role of CBF and oxygenation monitoring in prognostication after cardiac arrest and few studies suggest that guiding critical care post-resuscitation to mean arterial pressures above the minimal autoregulatory range might improve outcome. Important knowledge gaps thus remain in cerebral monitoring and CBF and oxygen goal-directed therapies post-resuscitation from cardiac arrest.

## Introduction

In the hours and days following successful resuscitation from cardiac arrest, various organs are recovering from global ischemia–reperfusion. The two key organs that determine the ultimate prognosis of pediatric patients resuscitated from cardiac arrest are the heart and the brain. Hypoxic ischemic encephalopathy persists and evolves during the days and weeks after cardiac arrest and is the major limiting factor in the full recovery of the victims of cardiac arrest. In the immediate period after cardiac arrest, there are important disturbances at the level of cerebral blood flow (CBF), cerebral oxygenation, and cerebral metabolism. The function of the neurovascular unit is disrupted. Despite great advances in the understanding of the cerebral pathophysiology post-resuscitation and several successful therapeutic advances in animal models, presently there are no therapies proven to be beneficial for improving neurological outcome in pediatric patients after cardiac arrest. Moreover, brain metrics are currently not routinely assessed after cardiac arrest.

The goal of this review is to reveal the knowledge gaps regarding post-resuscitation CBF disturbances in pediatric and adult patients, and ultimately to stimulate the development of cerebral goal-directed therapies for cardiac arrest. We will present several methods used for assessment of cerebral perfusion, and review the evolution of CBF during the after cardiac arrest first in animal models and then in humans.

## Methods Used for Evaluation of Cerebral Perfusion

Multiple methods are available for the assessment of CBF and cerebral perfusion. Some methods are strictly limited to use in animal models, while others may also be utilized at the bedside. Table 1 summarizes the various methods available for evaluation of cerebral perfusion in animals and humans.

**Table 1:** Current methods for assessment of cerebral blood flow (CBF) in animals and humans.

Method	Animals vs. humans	Invasive vs. non-invasive	Area measured	Serial vs. one time point	Comments
Laser Doppler	Animals	Invasive	Regional/small area	Serial	Measures perfusion in a localized area around the probe
Microspheres	Animals	Invasive	Regional	One time point	Requires sacrifice of the animal for analysis of blood flow
^14^C autoradiography	Animals	Invasive	Regional	One time point	Requires sacrifice of the animal for analysis of blood flow
Laser Speckle Flow	Animals	Invasive	Regional	Serial	Assesses cortical areas only. Requires deflection of the scalp and craniotomy in adult animals
*In vivo* multiphoton microscopy	Animals	Invasive	Regional/small area	Serial	Assesses perfusion in several areas of 50–100 µm over a 2 mm cortical window
Xenon CT	Animals	Non-invasive	Regional	Serial	Used for humans exclusively in research
Arterial spin labeling magnetic resonance imaging, PET, SPECT	Animals and humans	Non-invasive	Regional	Serial	Current gold standard for CBF measurements
Transcranial Doppler ultrasound	Animals and humans	Non-invasive	Regional/small area	Serial	Measures velocity at the level of middle cerebral artery. Indirect assessment of cerebral microvasculature
Near-infrared spectroscopy	Animals and humans	Non-invasive	Regional/small area	Serial	Measures tissue oxygenation in a localized area under the sensor
Thermal Diffusion flowmetry	Animals and humans	Invasive	Regional/small area	Serial	Utilized in humans after traumatic brain injury

### Methods for CBF Assessment in Animal Models

Various methods of CBF assessment have been used in animal models of cardiac arrest. A review of these methods is important to appreciate the benefits and additional information that can be acquired from research in animal models, as well as to aid in interpretation of blood flow data considering the limitations of each method. In animal models of cardiac arrest, available methods for the assessment of cerebral perfusion may be categorized regarding brain tissue penetration (invasive or non-invasive), and further categorized regarding the area assessed (regional vs. global), and time post-resuscitation (one time point vs. serially) (Table 1).

#### Invasive and Minimally Invasive Methods of Quantification of Cerebral Perfusion in Animal Models

Assessment of CBF can be performed invasively using intraparenchymal Laser Doppler probes (1), intravenously injected microspheres (2) or ^14^C iodoantipyrene autoradiography (3), laser speckle flowmetry (4, 5), and optical imaging (6, 7). Laser Doppler probes are inserted locally and can serially assess cerebral perfusion in a small area limited to the region surrounding the probe. Intravenously injected microspheres and autoradiography methods require harvesting of the brain to quantify the concentration of microspheres or intensity of the tracer. Thus, these methods can assess CBF at only one time point after cardiac arrest, but have the advantage of providing regional maps of CBF for various brain regions. Laser speckle flowmetry is a minimally invasive method for assessment of serial perfusion. In pediatric and neonatal aged rodents the scalp is incised and deflected and imaging is performed though the intact skull allowing perfusion measurements for large cortical areas, whereas in adult rats a craniotomy is often necessary and perfusion can be measured over an area of a few millimeters. At a microscopic level, the novel optical technologies of dark field imaging and *in vivo* multiphoton microscopy allow assessment of perfusion in animal models from the pial surface to cortical depths of 100–400 µm over a small area of a few millimeters. These techniques provide detailed analysis of the cortical microcirculation and allow for measurement of microvessel diameter, red blood cell speed and density, plasma transit time in the microcirculation, cell interactions at the neurovascular unit, and important assessments of the effect of clinically relevant as well as novel therapeutic agents on the microcirculation (7, 8).

#### Non-Invasive Methods for Use in Animal Models

Xenon CT (9), arterial spin labeling magnetic resonance imaging (ASL-MRI) (10), TCD, positron emission tomography (PET), single-photon emission computed tomography (SPECT), and dynamic susceptibility contrast magnetic resonance imaging (DSC-MRI) allow non-invasive measurement of perfusion. These methods have the ability to assess regional CBF at multiple time points after cardiac arrest. Details on these techniques are provided in the following section.

### Methods for CBF Assessment in Humans

Limited evaluation of CBF has been performed in humans after CA, due to the availability of only a few non-invasive means and portable devices. For human use, an ideal tool for CBF assessment post-resuscitation should be employable early post-resuscitation and be portable, non-invasive, not interfere with clinical care, and allow for serial assessments. Post-resuscitation research is still in search of this tool, and this represents the largest knowledge gap and the greatest impediment to goal-directed cerebral resuscitation.

Cerebral perfusion can currently be assessed in children and adults after cardiac arrest using three techniques: TCD, ASL-MRI, or Xenon CT. Additionally, implantable CBF monitors such as thermal diffusion flowmetry are available invasive methods which have been used to assess perfusion in isolated case reports of cardiac arrest (11) and have been extensively used in patients with traumatic brain injury and subarachnoid hemorrhage (12, 13). In the past, valuable CBF data were obtained in adults using tracers (^133^Xe) (14) or thermodilution methods (15); however, with the advent of the newer, less invasive technologies enumerated above, these two techniques have not been used recently.

#### Transcranial Doppler

Transcranial Doppler may be used immediately after cardiac arrest. It can provide serial assessment of cerebral perfusion, does not interfere with clinical care, and provides immediate results. TCD measures cerebral perfusion by assessing blood flow in the middle cerebral artery (MCA). It does not directly assess microvascular perfusion, although several parameters can be used to infer the status of cerebral microcirculation, especially in a global cerebral insult such as cardiac arrest. TCD uses a sonographic probe that is placed on the temporal area anterior to the ear and detects changes in the frequency of sound waves deflected by intravascular erythrocytes. Velocity, direction, and presence of blood flow in the MCA artery may be obtained using TCD (16, 17). As blood flow velocity in the MCA can increase due to local factors (vasoconstriction) or due to increased cardiac output, the ratio between MCA velocity and intracranial carotid artery (ICA) velocity [Lindegaard ratio (LR)] is used to differentiate between cerebral vasoconstriction and decreased cardiac output. Cerebral vasospasm or vasoconstriction will increase the MCA velocity while the ICA velocity would be unaffected, resulting in an increased LR > 3, whereas increased cardiac output would increase both MCA and ICA velocities, resulting in LR < 3 (17). A useful index for measuring microvascular perfusion is the pulsatility index (PI), which takes into account the systolic and diastolic velocities in the MCA and the mean flow velocity to differentiate between normal (PI = 0.6–1.1), low resistance conditions (PI < 0.6, hyperemia vs. vasospasm vs. stenosis), high resistance (PI = 1.2–1.6, in case of microvascular derangements or mild increase in intracranial pressure), or very high resistance (PI = 1.7–1.9, severe increase in ICP), or absent CBF (PI = 2, cerebral asystole) (18). TCD is an available tool for assessment of cerebral perfusion, and it is currently underutilized in clinical care post-resuscitation.

#### Arterial Spin Labeling Magnetic Resonance Imaging

Arterial spin labeling magnetic resonance imaging is currently the gold standard technique for assessment of CBF (19). It provides data on regional CBF, and it allows for correlation with cerebral metabolism if nuclear magnetic resonance spectroscopy is acquired in the same patient. ASL-MRI requires patient transport to the MRI suite, it cannot be done in the presence of metal devices, and the scan time is 30–60 min. Therefore, ASL-MRI is not feasible during the early period after resuscitation, as patients require intense monitoring, titration of blood pressure medication, and may undergo other various therapies such as therapeutic hypothermia. In two recent studies, ASL-MRI was performed at 6 ± 4 days after cardiac arrest in one study, and in another at a median time of 5 days, range of 1.6–10.4 days after cardiac arrest (20, 21). Future advancement in ASL-MRI scanning techniques to shorten the scan time, availability of MRI scanners in close proximity to intensive care units, and a move toward universal MRI safe equipment for critically ill patients may allow in the future obtaining MRI images to assess structure, perfusion, and metabolism after the initial stabilization post-resuscitation.

#### Xenon Computer Tomography (Xe-CT)

Xenon computer tomography is another method of quantitative CBF measurement, which could theoretically be used at the bedside. The Xe-CT methods detect the distribution of the tracer in the brain. When the method was initially developed, Xe^133^ was injected in the carotid artery, and CBF was calculated from its clearance curve (22). Subsequently, the intracarotid Xe method has been replaced with the less invasive Xe^133^inhalation method and Xe-enhanced CT. Compared with Xe^133^ technique, the Xe-enhanced technique avoids extracerebral contamination, has higher spatial resolution, and it has been used in both human and animals post-resuscitation as early as 8 h after resuscitation (9, 23). Xe-CT scan is portable and has relatively safe profile, however, the inhalation of Xe at higher concentration has the potential risk of respiratory depression and increased ICP (24). In addition, only a few pediatric intensive care units worldwide have Xe-CT available for use, and to our knowledge Xe-CT is currently used strictly for research purposes.

#### Near-Infrared Spectroscopy (NIRS)

Near-infrared spectroscopy measures the oxygen saturation in tissues using the differences of near-infrared (700–1,000 nm) light absorption between oxygenated and deoxygenated hemoglobin. The intensity changes between the transmitted and received NIR light are detected by a sensor and used to determine the tissue oxygen saturation (25). NIRS can be used to assess relative brain oxygenation and is considered a surrogate of CBF (26); it is relatively easy to use and it does not interfere with clinical care. Its limitations thus include significant attenuation at the extracerebral tissue, limited depth penetrance with measurement limited to the cortex, and lack of absolute measurement values that can be compared directly across subjects and conditions (25). Longitudinal assessment of NIRS post-resuscitation might signal changes consistent with clinical deterioration or might direct clinical care by establishing an optimal blood pressure for cerebral perfusion (27). NIRS can also be used to correlate changes in cerebral oxygenation with blood pressure to assess blood pressure autoregulation (27, 28).

In summary, multiple methods for assessment of CBF and cerebral perfusion are available in animals, and with the recent addition of novel methods such as optical imaging detailed assessment of the effect of critical care interventions and clinically relevant therapies on CBF in animal models is possible. Detailed characterization of regional CBF post-resuscitation has been ascertained from animal models, yet only limited data on post-resuscitation CBF is available for humans, due to the availability of only a few methods for clinical assessment of CBF. Partnering with engineering teams to design devices for bedside cerebral monitoring after cardiac arrest is necessary for assessment of cerebral function and perfusion, and ultimately to conduct cerebral resuscitation.

## Alterations in CBF after Cardiac Arrest: Animal Models

Cardiac arrest produces a global cerebral ischemic injury. Early animal models of global cerebral ischemia included, among others, aortic balloon occlusion (29) and neck tourniquet insults (30). Clinically relevant models of cardiac arrest were developed more recently, and CBF was thoroughly assessed using these models. These models include: ventricular fibrillation cardiac arrest (VF) (31), asphyxial cardiac arrest (10, 31), and KCl-induced cardiac arrest (32). CBF has been characterized after cardiac arrest in pediatric and adult age groups in rats, pigs, cats, and rabbits. Animal models offer the advantage of serial and regional assessment of CBF providing a temporal frame of the cerebrovascular changes in the four phases of post-cardiac arrest syndrome: immediate (0–20 min), early (20 min to 6–12 h), intermediate (6–12 to72 h), and recovery (>72 h) (33). Another benefit of the animal models is the ability to assess CBF after cardiac arrest of progressive durations, which provides important clinical insight for alterations of CBF in insults of moderate duration (in-hospital cardiac arrest model) vs. prolonged durations (out-of-hospital cardiac arrest model). Finally, the ability to correlate CBF and CBF-related interventions with functional outcome and histology is another important aspect of experimental cardiac arrest models.

Classically, three stages of CBF alterations have been described after resuscitation from cardiac arrest. During cardiac arrest and CPR, there is initially absence of flow during cardiac arrest and subsequently either no-flow or low-flow perfusion during CPR. After return of spontaneous circulation, the initial post-resuscitation CBF stage is known as cerebral hyperemia, and occurs between 5 and 30 min after resuscitation. Following hyperemia, cerebral hypoperfusion occurs from 30 min to 6 h after resuscitation. Finally, either resolution of CBF, continued hypoperfusion, global hyperemia, or cessation of flow and brain death occurs during the days post-cardiac arrest (34). More recent studies, however, reveal that cerebral perfusion during each stage is different in various brain regions: subcortical areas generally display early hyperemia, whereas cortical areas generally display hypoperfusion. Additionally, cerebral perfusion is dependent on insult duration: hyperemia is less frequently observed in prolonged insults, whereas hypoperfusion is more pronounced in prolonged insults. Finally, the age of the animal and the type of cardiac arrest also influence post-resuscitation CBF. Immature animals have different hemodynamic response to stimuli due to ongoing neurogenesis, synaptic pruning, and changes in the vascular architecture of the brain (35, 36).

Significant data have been gathered on CBF changes in animal models during each of the four phases of the post-cardiac arrest syndrome. We present their highlights below.

### CBF in the Immediate Phase of the Post-Cardiac Arrest Syndrome (0–20 min)

In the immediate phase post-resuscitation, CBF disturbances have traditionally been characterized by hyperemia; however, recent studies show that the alterations are brain-region specific depend on three factors: age (pediatric vs. adult model), pathophysiological mechanism of cardiac arrest (VF vs. asphyxia), and insult duration (mild, moderate vs. severe insults).

In the adult experimental cardiac arrest, hyperemia is repeatedly noted in various models. Blomqvist and Wieloch (37) reported hyperemia of different intensities in 17 regions early after VF cardiac arrest in rats. Similarly, early hyperemia was also found in adult rats and dogs with longer duration (10–13 min) of VF cardiac arrest (38, 39). Hyperemia after asphyxial cardiac arrest was also described by Xu et al. in the thalamus and cortex (40). The regional distribution of hyperemia was also recognized in a dog model of VF cardiac arrest: the brain stem and basal ganglia had longer duration of hyperemia compared with the cortex (41). A comprehensive comparison of CBF in VF cardiac arrest vs. asphyxial cardiac arrest in adult rats revealed that after asphyxial cardiac arrest marked early hyperemia is observed in all regions, whereas after VF cardiac arrest early hyperemia was limited to the cortex, whereas subcortical regions had baseline levels of CBF (31). The influence of insult duration on CBF has not been completely characterized in adult models of cardiac arrest. To date, no studies provided a parallel assessment of regional CBF in insults of progressive durations in models of adult cardiac arrest.

**Figure 1:**
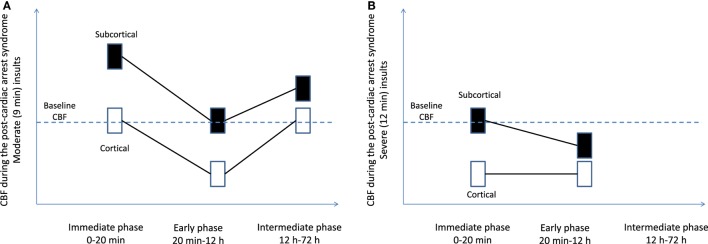
Regional cerebral blood flow (CBF) after pediatric asphyxial cardiac arrest after moderate **(A)** and prolonged **(B)** insults. Figure is a graphic representation of a compilation of two studies from our laboratory (10, 55).

In pediatric asphyxial cardiac arrest in rats, CBF is markedly dependent on the brain region (cortical vs. subcortical) and insult duration (moderate vs. severe insult) (Figure 1) (10). In contrast with earlier studies in adult animal models showing universal early hyperemia, in immature rats hyperemia is limited to the subcortical areas, whereas cortical areas have normal or even decreased CBF. Specifically, in insults of moderate duration (9-min asphyxia) subcortical areas are characterized by marked early hyperemia. However, after insults of prolonged duration (12 min) subcortical areas have baseline CBF levels without evidence of hyperemia (Figure 1) (10). Cortical CBF is markedly different than subcortical CBF. Cortical CBF returns to baseline level immediately after resuscitation in insults of moderate duration, whereas after cardiac arrest in insults of prolonged duration cortical hypoperfusion occurs immediately post-resuscitation (Figure 1) (10). Measurement of brain tissue oxygen level in these insults revealed that brain tissue oxygen mirrored the CBF changes: subcortical hyperoxia occurred in insults of moderate duration, while cortical hypoxia occurred in insults of severe duration (42). These studies in pediatric models of cardiac arrest showed that alterations in CBF are accompanied by alterations of tissue oxygenation post-resuscitation.

The significance of early hyperemia is still being elucidated. Some studies suggest that hyperemia is an indication of coupling of CBF and metabolism, and thus is an indication of increased local metabolic rates. Others suggest that hyperemia is detrimental as it intensifies the reperfusion syndrome, and propose that a more gradual reperfusion might be beneficial. Thalamic areas, where early hyperemia is observed after pediatric cardiac arrest, is characterized by extensive degeneration of neurites and activation of microglia, suggesting an association between early hyperemia and neurodegeneration (43). Therapies targeting the early hyperemic phase to improve neurological outcome included antioxidants such as superoxide dismutase and polynitroxyl albumin, which decreased the early hyperemia of the subcortical areas and improved outcome (10, 44).

### CBF in the Early Phase of Post-Cardiac Arrest Syndrome (20 min–12 h)

After the initial period post-resuscitation, CBF is generally characterized by a longer hypoperfusion stage, which appears to occur more uniformly across species, age groups, and models (34, 37–39, 41, 45–47). Hypoperfusion is observed anywhere from 15 to 60 min post-resuscitation and can persists for hours or even days.

In adult cardiac arrest models, both asphyxial and VF, hypoperfusion has been observed in all regions, cortical and subcortical (31). Gray matter appeared to be more vulnerable to hypoperfusion than the white matter (48).

In pediatric asphyxial cardiac arrest in immature rats the hypoperfusion is more evident in the cortex and is more pronounced in longer durations of cardiac arrest (10, 49). In the subcortical areas such as thalamus, hypoperfusion was seen only with prolonged durations of cardiac arrest (10) (Figure 1).

Cerebral blood flow and metabolism are likely uncoupled during the delayed hypoperfusion period, suggested by the cortical hypoxia observed after pediatric asphyxial cardiac arrest (42), and suggesting that the brain suffers a secondary ischemic event during this period. Multiple mechanisms are implicated in the development of delayed hypoperfusion, including damage at the level of the endothelial cells, unbalance of local vasodilator and vasoconstrictors, as well as impaired autoregulation in the setting of decreased blood pressure. Therapies targeting these mechanisms have been assessed in animal models (5, 30, 50–52).

### CBF in the Intermediate Phase of the Post-Resuscitation Syndrome (12–72 h)

Cerebral blood flow at greater than 12 h after experimental cardiac arrest has not been extensively studied. In severe cardiac arrest models, the animals need intensive care and might not survive if extubated and returned to their cages. CBF was assessed at 24 h after moderate durations of pediatric cardiac arrest (9 min, Figure 1). CBF returned to normal values in the delayed period in all regions except for the thalamus. Thalamic areas have increased CBF at 24 h after CA (53). The function of thalamic circuitry is impaired after pediatric cardiac arrest, as demonstrated by increased firing rates in thalamocortical neurons at 48–72 h after cardiac arrest and associated with histologic evidence of injury in the thalamic nuclei.

### Cerebral Perfusion at the Microvascular Level: No-Reflow Phenomenon after Cardiac Arrest

During the immediate and early periods after cardiac arrest there are microcirculatory disturbances, resulting in localized areas of no flow, interspersed with areas of low flow and increased flow. This phenomenon was initially described by Ames et al. in a model of global ischemia in rabbits in 1968 as incomplete filling of the cortical capillary bed upon perfusion with contrast agent and is known as the no-reflow phenomenon (54). The perfusion deficits increased with increased duration of ischemia and the no-reflow phenomenon has been observed in various models of cardiac arrest (55, 56).

Multiple mechanisms have been proposed to be responsible for the no-reflow phenomenon, including post-resuscitation hypotension, increase in blood viscosity, and fibrin clots (34, 57–59). Therapies targeting these mechanisms have shown beneficial effect in animal models. For example, blood flow promotion therapy with hypertension and hemodilution immediately after cardiac arrest has been shown to normalize the CBF pattern and improve outcome in dogs (46, 60). Thrombolytic therapies with plasminogen activator and heparin reduced no-reflow phenomenon seen after cardiac arrest and produced more homogeneous perfusion (61). The no-reflow phenomenon likely contributes to the hypoperfusion observed post-resuscitation and likely causes a secondary ischemic injury.

In summary, valuable data regarding alterations of CBF post-resuscitation is obtained from animal models of cardiac arrest. These models have the advantage of allowing serial, regional assessment of CBF, and comparison of CBF alterations in insults of progressive durations, simultaneous assessment of CBF, brain oxygenation, metabolism, and electrical activity (62), assessment of microvascular alterations, and development of novel CBF and neuronal targeted therapies.

## CBF Dysregulation after Cardiac Arrest in Children and Adults

Data on CBF from animal models provide us with an appreciation of post-cardiac arrest perfusion variability for different types of cardiac arrest (asphyxial vs. VF), and progressively longer insults in pediatric and adult models. CBF data from humans are generally compiled from various insult types (VF, asphyxia, and often unknown), and various and unknown insult durations. Thus, human CBF data post-cardiac arrest are more variable. The vast majority of CBF data after cardiac arrest have been ascertained from adults and have been obtained at delayed time points post-resuscitation. A prevailing pattern of CBF post- resuscitation is the protracted hypoperfusion followed by hyperemia in the days following cardiac arrest. The current CBF data demonstrate the presence of perfusion abnormalities after cardiac arrest and underscore the need for cerebral monitoring post-resuscitation.

Cerebral perfusion in children varies greatly with age paralleling cerebral maturation during development, and thus combining data from different age groups in the pediatric population may be a confounding factor (63). Highlighting the dynamic nature of brain development in infancy, childhood, and adolescence, global CBF values average 16 ml/100 g/min in the neonate, increase to 39 ml/100 g/min in infancy, further increase to 100 ml/100 g/min by 6–8 years of age, and finally decrease to 60 ml/100 g/min in males and 75 ml/100 g/min in females by age 18 (63–67). Within each age group, some individual variation exists and regional differences in CBF at each age group are noted. The basal ganglia and the thalamus have the highest perfusion values, and the gray matter has lower perfusion values (67). Because of individual and age-related variations, age-matching is imperative in studies related to cerebral hemodynamics. Comparing mean CBF values at one time point post- resuscitation between groups of children of wide age ranges is unlikely to lead to interpretable results. Ideally, although difficult, repeated longitudinal assessment of perfusion in the same subject and correlation with metabolism and tissue oxygenation would be performed.

We will present below the current knowledge on CBF post-cardiac arrest in adult and pediatric patients assessed by TCD, ASL-MRI, Xe-CT, and CBF autoregulation after cardiac arrest (Table 2).

**Table 2:** Summary of studies assessing cerebral blood flow (CBF) post-resuscitation.

Reference	Method	Age	Time point	Highlights
Iida et al. (68)	Transcranial Doppler (TCD)	Adults	0–12 h	Low mean flow velocity, high pulsatility index (PI). Suggestive of microangiopathy, vasoconstriction, and no reflow
Lemiale et al. (69), Buunk et al. (70)	TCD	Adults	12–24 h	Low mean flow velocity, high PI. Suggestive of microangiopathy, vasoconstriction, and no reflow
Doepp Connolly et al. (71)	TCD	Adults	0–48 h, 3–5 days, 7–10 days post-resuscitation	No correlation of TCD and outcome
Lin et al. (72)	TCD	Children	Before, during, and after hypothermia	Undetectable flow was associated with death. Markers for good prognosis: normal flow velocity during rewarming and normal PI during rewarming or hypothermia
Manchester et al. (21)	Arterial spin labeling magnetic resonance imaging (ASL-MRI)	Children	>24 h post-resuscitation	No CBF difference between patients with favorable and unfavorable outcomes. MRI was performed at 6 ± 4 days after cardiac arrest
Pollock et al. (73)	ASL-MRI	Adults and children	1–13 days post-resuscitation	Global hyperperfusion pattern identified. Most patients had poor prognosis
Beckstead et al. (75)	^133^Xenon Washout	Adults	2–6 and >6 h post-resuscitation	Decreased CBF and decreased oxygen metabolism at 2–6 h, followed by increased CBF with relative hyperemia at >6 h
Cohan et al. (76)	Xenon inhalation	Adults	18–36 h post-resuscitation	Increased CBF was associated with coma, isoelectric encephalogram, and death. Normal CBF was associated with regaining consciousness
Brodersen (77)	Xenon inhalation	Adults	1–12 days post-resuscitation	Most patients had relative hyperemia. CBF was variable and paralleled the oxygen metabolism
Sundgreen et al. (78)	TCD	Adults	0–24 h post-resuscitation	Cerebral autoregulation was either absent or right-shifted
Nishizawa and Kudoh (79)	CBF index	Adults	3 days post-resuscitation	All patients were comatose. Impaired cerebral autoregulation was detected

### Cerebral Perfusion Assessment in Adults and Children Using TCD

Transcranial Doppler was used in several studies after cardiac arrest. These studies confirmed that cerebral hemodynamics are altered during the post-cardiac arrest syndrome and evolve during the first 24–72 h after cardiac arrest.

During the first 12 h after cardiac arrest several small studies in adults demonstrate the presence of hypoperfusion and high vascular resistance, evidenced by low mean flow velocity and high PI (68–70). This diffuse hypodynamic TCD pattern suggests microangiopathy secondary to vasoconstriction and no-reflow in the microcirculation, and is associated with poor neurological outcome (18).

After 24 h post- resuscitation, a diffuse hyperdynamic pattern is observed, evidenced by high mean flow velocity and low PI, suggesting hyperemia or vasospasm (68–70). This pattern was also associated with poor prognosis, progression to brain death, and increased ICP (18). These changes were associated with increased endothelin levels, decreased nitric oxide levels, and increased cGMP levels, suggesting that an imbalance between local vasodilators and vasoconstrictors plays a role in the cerebral vasoconstriction and hypoperfusion phase (70).

Underscoring the importance of serial assessment of CBF post-cardiac arrest, one study showed that the initial hypodynamic pattern observed during the first 12 h after resuscitation was followed by normalization of mean flow velocity and PI for 24 h, and then by increased mean flow velocity and low PI for 48–72 h (69). Therefore, a single measurement cannot be used for prognostication without taking into account the CBF trajectory for the respective patient. Possibly related to the time of measurement of CBF post-resuscitation, a recent study failed to show a correlation between TCD parameters and outcome. In this particular study, the first CBF measurement was obtained within 48 h after cardiac arrest, and subsequent measurements were obtained at days 3–5 and 7–10 post-resuscitation (71).

Transcranial Doppler was successfully assessed in 17 children after cardiac arrest at three points: during the pre-hypothermia, hypothermia, and rewarming phases. All patients with undetectable flow during any phase died. Patients with normal mean flow velocity during the rewarming phase had better prognosis vs. patients with low mean flow velocity. Patients with normal pulsatility indices during the hypothermia and rewarming phases had better outcomes compared with the ones with high pulsatility indices (72).

### CBF Assessment in Adults and Children Using ASL-MRI

MRI assessment of CBF early after cardiac arrest is difficult to perform, due to the patients’ clinical instability. In a prospective observational study, pediatric patients underwent MRI to assess CBF and ADC maps after cardiac arrest (21). This study was the first pilot study in the pediatric patients to assess CBF after cardiac arrest and identified both the opportunities and current challenges of ASL-MRI after cardiac arrest. The patients were transported to the MRI suite when the clinical team was comfortable that the patient was stable for transport and seizure-free. The brain MRI was performed at 6 ± 4 days after cardiac arrest (IQR 2.7–8.7 days).

Cerebral blood flow was 76.8 + 32.5 vs. 91.6 + 38.9 ml/100 g/min for patients with favorable vs. unfavorable outcome, with no significant difference between patients with favorable vs. unfavorable outcomes. Importantly, children with unfavorable outcome had decreased apparent diffusion coefficient compared with children with favorable outcome, suggesting cytotoxic edema. Brain regions with abnormalities in diffusion consistent with cerebral edema had also increased CBF. The assessment of CBF at only one time point after cardiac arrest and the necessity to analyze data after cardiac arrest among patients of different ages could have contributed to the limited conclusions that could be drawn regarding CBF from this study. Another study of 14 adults and 2 children that underwent ASL-MRI at a range of 1 and 13 days after cardiac arrest showed a universal global hyperperfusion pattern. A majority of patients in this latter study died (10/14 adults, and 2/2 children) (73).

### CBF Assessment in Adults Using Inhalation of Xe^133^ and the Ketty-Schmidt Technique

Before the advent of MRI, CBF was measured in human subjects for decades using the Xe^133^ washout technique. Xe^133^ was administered by inhalation, and CBF was calculated using a compartment method (74). With this technique, human CBF and metabolism were assessed in eight adult patients post-resuscitation with inhalation of Xe^133^ and concomitant measurement of CMRO_2_ using the jugular venous oxygen tension measurement technique. From 2 to 6 h post-cardiac arrest, CBF was decreased to 50% along with a decrease in oxygen metabolism. After 6 h, CBF was increased toward baseline, while the metabolism also started recovering, albeit disproportionately in comparison to CBF, such that a relative hyperemia developed. This “luxury perfusion” was also seen at 24–60 h after cardiac arrest. This study suggested that CBF at delayed time points after cardiac arrest is uncoupled from metabolism (75).

Similarly, in 13 patients resuscitated from cardiac arrest, CBF was assessed using Xe inhalation. Seven (54%) patients who regained consciousness had normal CBF values, while six (46%) patients who did not regained consciousness had increased CBF between 18 and 36 h. In these comatose patients, decreased CBF after the period of hyperemia was associated with isoelectric encephalogram and death (76). In another similar study, a majority of patients had relative hyperemia; however, CBF showed great variability between patients (77).

### Assessment of CBF Autoregulation and CO_2_ Reactivity after CA

Cerebral blood flow autoregulation is a physiological phenomenon that maintains a relatively constant CBF for blood pressures in the range of 50–150 mmHg, through vasodilatory and vasoconstrictor mechanisms.

After cardiac arrest, CBF autoregulation was found to be compromised in 13 of 18 adult patients in the first 24 h after resuscitation. For assessment of autoregulation, the blood pressure was increased with 30 mmHg using intravenous infusion of norepinephrine. The autoregulation was absent in some patients, while the lower limit of autoregulation was shifted toward a higher pressure in others. Five patients (28%) had normal autoregulation, eight patients (45%) had loss of autoregulation, and another five patients (28%) had preserved but right-shifted autoregulation, with the lower limit of autoregulation increased to a median of 114 mmHg (range 80–120), a derivation from a median of 76 mmHg (range 41–105) in the healthy individuals studied (78). Cerebral autoregulation was also found to be impaired in eight adult patients that were comatose 3 days after cardiac arrest. The patients had internal jugular vein cannulation, and CBF index was indirectly calculated using the arterial-jugular bulb venous oxygen content difference (79).

More recently, NIRS was validated as a tool for measuring cerebrovascular autoregulation (80). Correlating changes in oxygen saturation with fluctuations of arterial blood pressure over time can detect an impairment in the CBF autoregulation. Several studies correlated impaired autoregulation with worse neurological outcome after cardiac arrest. In a study in adults a combined measure of tissue oxygenation and blood pressure was performed daily for the first 3 days after CA. Impaired autoregulation on days 1–3 after cardiac arrest was associated with increased mortality at 3 months (81). In another study, impaired autoregulation was found in 35% patients during the first 24 h after cardiac arrest and was correlated with worse outcomes (82). Since hypotension after cardiac arrest was shown to be deleterious and higher blood pressure after cardiac arrest were associated with favorable outcomes (83, 84), it is important to maintain the blood pressure in an optimum range for cerebral perfusion. Important studies by Lee et al. generated an index of vasomotor reactivity by correlating relative tissue hemoglobin with mean arterial pressure (MAP), and based on this index, the optimal MAP range with most robust autoregulation can be identified (28, 85). In a landmark pilot study in 35 pediatric patients monitored with NIRS after cardiac arrest, patients who spent more time with MAP below the optimal autoregulatory range during the first 48 h after cardiac arrest had worse outcomes (27). Thus, combining real-time NIRS with continuous blood pressure monitoring might not only be used as a tool for prognostication early after cardiac arrest, and may serve as a measure of goal-directed blood pressure management after cardiac arrest.

The cerebrovascular reactivity to changes in arterial carbon dioxide tension (CO_2_ reactivity), accountable for a 3% decrease in CBF in response to a 1 mmHg decrease in pCO_2_ (86), appears to be maintained after cardiac arrest (70). Thus, hyperventilation after cardiac arrest might induce cerebral vasoconstriction and a secondary ischemic insult and should, therefore, be avoided.

In summary, there are relatively few studies assessing CBF post-resuscitation in adults, and even fewer studies in children. CBF varies greatly from infancy to adolescence, and thus pediatric studies of CBF should group data for each age group (neonate, infant, toddler, school age, and adolescent) to allow for meaningful interpretation of results. The limiting factor for assessing CBF after cardiac arrest is a reliable non-invasive device that can be used at the bedside and allow for serial measurements of cerebral perfusion. TCD could offer non-invasive repeated measures of cerebral perfusion, and is largely underutilized after cardiac arrest. There are good data to support that decreases in CBF correlate with decreases in cerebral oxygenation (1, 42) and thus NIRS could provide some guidance post-resuscitation especially to guide maintenance of blood pressure within the range with most robust autoregulation (27).

In conclusion, we reviewed here CBF changes in animal models and in adult and pediatric patients after cardiac arrest. There are many challenges to the assessment of perfusion after cardiac arrest. Multidisciplinary and multi-institutional collaborations are necessary to fully evaluate CBF dysregulation in pediatrics, as CBF varies vastly in an age-dependent manner. Understanding cerebral perfusion after cardiac arrest would be beneficial in guiding current therapies, assessing novel vasoactive therapies, and prognostication.

## Author Contributions

Conception and design; critical revision of the article for important intellectual content; final approval of the article: MM, BI, LL, and RC. Drafting of the article: LL, BI, and MM.

## Conflict of Interest Statement

The authors declare that the research was conducted in the absence of any commercial or financial relationships that could be construed as a potential conflict of interest.
